# Study of RNA interference inhibiting rat ovarian androgen biosynthesis by depressing 17alpha-hydroxylase/17, 20-lyase activity in vivo

**DOI:** 10.1186/1477-7827-7-73

**Published:** 2009-07-17

**Authors:** Yi Li, Xiao-yan Liang, Li-na Wei, Yong-lao Xiong, Xing Yang, Hui-gan Shi, Zi-hong Yang

**Affiliations:** 1 Center for Reproductive Medicine, First Affiliated Hospital, Sun Yat-sen University, Guangzhou, PR China; 2 Daan Gene Diagnosis Center, Sun Yat-sen University; Guangzhou, PR China

## Abstract

**Background:**

17alpha-hydroxylase/17, 20-lyase encoded by CYP17 is the key enzyme in androgen biosynthesis pathway. Previous studies demonstrated the accentuation of the enzyme in patients with polycystic ovary syndrome (PCOS) was the most important mechanism of androgen excess. We chose CYP17 as the therapeutic target, trying to suppress the activity of 17alpha-hydroxylase/17, 20-lyase and inhibit androgen biosynthesis by silencing the expression of CYP17 in the rat ovary.

**Methods:**

Three CYP17-targeting and one negative control oligonucleotides were designed and used in the present study. The silence efficiency of lentivirus shRNA was assessed by qRT-PCR, Western blotting and hormone assay. After subcapsular injection of lentivirus shRNA in rat ovary, the delivery efficiency was evaluated by GFP fluorescence and qPCR. Total RNA was extracted from rat ovary for CYP17 mRNA determination and rat serum was collected for hormone measurement.

**Results:**

In total, three CYP17-targeting lentivirus shRNAs were synthesized. The results showed that all of them had a silencing effect on CYP17 mRNA and protein. Moreover, androstenedione secreted by rat theca interstitial cells (TIC) in the RNAi group declined significantly compared with that in the control group. Two weeks after rat ovarian subcapsular injection of chosen CYP17 shRNA, the GFP fluorescence of frozen ovarian sections could be seen clearly under fluorescence microscope. It also showed that the GFP DNA level increased significantly, and its relative expression level was 7.42 times higher than that in the control group. Simultaneously, shRNA treatment significantly decreased CYP17 mRNA and protein levels at 61% and 54%, respectively. Hormone assay showed that all the levels of androstenedione, 17-hydroxyprogesterone and testosterone declined to a certain degree, but progesterone levels declined significantly.

**Conclusion:**

The present study proves for the first time that ovarian androgen biosynthesis can be inhibited by silencing CYP17 expression. It may provide a novel strategy for therapy of hyperandrogenism diseases, and also set an example for the use of RNAi technology in endocrine diseases.

## Background

Polycystic ovary syndrome (PCOS) is one of the most common gynecological endocrine diseases, which has an incidence rate of 5–10% in women of reproductive age. Hyperandrogenism is closely associated with PCOS, and is regarded as the most important presentation of PCOS. About 60–80% of patients with PCOS have signs of hyperandrogenism, such as hypertrichosis, acne and baldness [1-3]. Additionally, the patient with PCOS often presents with anovulation which is associated with follicular dysfunction induced by hyperandrogenism [4]. Androgen excess can also impair glucose tolerance, leading to insulin resistance, and causing a series of metabolic diseases [5,6]. Although some anti-androgen drugs (such as cyproterone metformin), single or in combination, have been used to treat the patient with PCOS, but therapeutic effects are unsatisfied and side effects are still under concerns [7,8].

17alpha-hydroxylase/17, 20-lyase is the key enzyme in androgen biosynthesis pathways. It resides in the ovary and the adrenal gland and is encoded by CYP17. This enzyme has double activities of 17alpha-hydroxylase and 17, 20-lyase, which are necessary for the conversion of pregnenolone to 17-hydroxypregnenolone and dehydroepiandrosterone, and for the conversion of progesterone to 17-hydroxyprogesterone and androstenedione. Several studies have demonstrated the accentuation of 17alpha-hydroxylase/17, 20-lyase as an important mechanism of androgen excess [9,10], in patients with PCOS. It was confirmed by an in vitro study of ovarian theca cells from a patient with PCOS, that the accentuation of 17alpha-hydroxylase/17, 20-lyase was caused by augmentation of CYP 17 at transcriptional levels [11,12].

RNA interference (RNAi) as an emerging biological technology for silencing gene expression has become a potentially powerful tool for therapy of clinical diseases. At present, outstanding progress has been made using RNAi in the therapy of tumors [13,14], viral infections [15-17] and genetic diseases [18-20]. It was reported that silencing Fas expression with RNAi holds therapeutic promise to prevent liver fulminant hepatitis [15]. Study also showed that silencing mutant SOD1 using RNAi could protect against neuro-degeneration and extend survival in an ALS model [18]. In our study, we chose CYP17 as the therapeutic target, trying to partly suppress the activity of 17alpha-hydroxylase/17, 20-lyase and inhibit rat androgen biosynthesis by silencing the expression of CYP17. We hope our research can provide a new direction for treatment of hyperandrogenism in the future.

## Methods

### Animals

Eighteen female Sprague-Dawley rats, aged two months and weighing 200 g, were raised in an animal laboratory of specific pathogen-free grade, with free access to food and water. Our study was approved by the ethics committee of the First Affiliated Hospital of Sun Yat-sen University. The animals were sacrificed by a lethal dose of chloral hydrate. The ovaries were removed for in vitro culture of theca interstitial cells (TIC) and preparation of frozen ovarian sections. Serum samples were stored at -20°C until use for hormone assays. During in vivo studies, the rats were divided into three groups of 6 rats: CYP17 RNAi group, negative control group, and blank control group.

### Design and construction of lenivirus CYP17 shRNA

**Figure 1:**
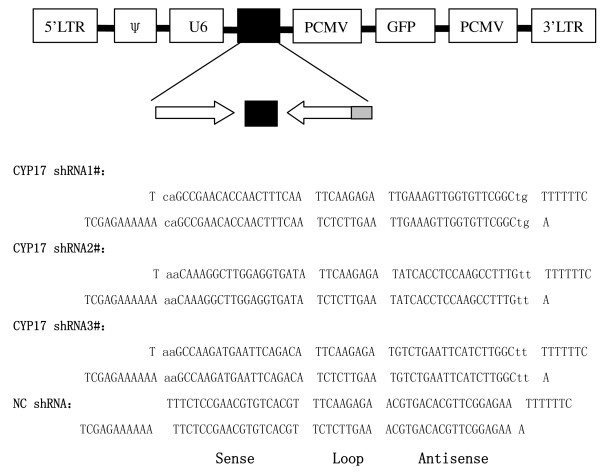
**Sequences of lentivirus CYP17shRNAs and NC shRNA**. NC: Negative Control.

Three CYP17-targeting oligonucleotides were designed, and another one was the negative control (Fig. 1). The stem loop DNA nucleotides were synthesized, and cloned into pGL-Lentivirus vectors by Genechem Co., Ltd (Shanghai, China). Lentivirus particles were prepared by Lentivirus Expression Systems (Shanghai, China) and viral titers were 5 × 10^8 ^transducing units/ml.

### Rat ovarian TIC isolation and culture

The rat ovarian TIC was isolated and purified with discontinuous percoll gradient centrifugation as described previously [21]. The TIC was cultured in a 12-well plate (Falcon, Becton Dickinson Labware, Lincoln Park, NJ) (1 × 10^6 ^cells/well). Each experiment was performed in a minimum of triplicate wells, and at least three experiments were performed in qRT-PCR, Western-blot and hormone assays to verify the silencing effect of constructed CYP17shRNAs in vitro. The culture medium was McCoy's5a (Invitrogen, CA) with supplementation of L-glutamine, BSA (1 mg/ml), penicillin (10000 IU/ml), and streptomycin (10000 mg/ml). Incubation was carried out at 37°C in humidified air with 5% CO_2 _for 5 or 7 days.

### Quantitative PCR

To analyze CYP17 mRNA levels, total RNA was extracted from cultured TIC or rat ovarian tissue, and used as a template for cDNA synthesis using oligo (dT) primers with the superscriptIII kit (Invitrogen, CA). Total DNA was extracted from the rat ovary to assess GFP DNA levels. Real-time quantitative PCR was performed using the ABI Prism 7500 detection system with SYBR green DNA detection kit (Applera, NY). The expression levels of housekeeping genes encoding β-actin were also quantified, and used for normalization. The PCR primers for CYP17 were as follows: Forward primers 5'-GGCCTTTGCAGATGCTGGTA-3', reverse primer 5'-GGAAAAGGTGCTGAACACCAA-3'. The PCR primers for β-actin were as follows: Forward primers 5'-GCTGCTGACCCCCACTGAT-3', reverse primer 5'-GCCACTGCCGGACAACTC-3'. The reactive condition of CYP17 PCR was 94°C 3 min, 94°C 30 sec, 55°C 60 sec, 10 cycles, then 94°C 30 sec, 55°C 60 sec, 30 cycles. The PCR primers for GFP were as follows: Forward primers TGCTTCAGCCGCTACCC, reverse primer CTTGCCGTAGTTCCACTTGA. The reactive condition of GFP PCR was 95°C 15 sec, 95°C 5 sec, 60°C 30 sec, 45 cycles in total.

### Western-blotting

Protein samples were extracted from cultured cells using cell lysis buffers. Each sample of protein (15 μg) was fractionated by SDS-PAGE. Following transfer onto a nitrocellulose membrane, blots were probed with a goat polyclonal antibody to CYP17 (1:1000, Santa Cruz Biotechnology, CA), and a rabbit polyclonal antibody to β-actin (1:1000, Santa Cruz Biotechnology). Blots were then incubated with anti-goat and anti-rabbit secondary antibodies (1:5000) tagged with horseradish peroxidase. All blots were visualized with Enhanced Chemiluminescence (Amersham Biosciences, Uppsala, Sweden) and analyzed by Image J Software (NIH, Maryland).

### Hormone assay

After collection, culture medium and rat serum samples were stored at -20°C for hormone measurement. Androstenedione and 17-hydroxyprogesterone levels were measured using an enzyme-linked immunosorbent assay kit (Diagnostic System Laboratories, TX). The detection limit of the assay was 0.05–10 ng/ml. The intra- and inter-assay coefficients of variation were 5 and 8.0%, respectively. Testosterone and progesterone levels were measured using electrochemiluminescence immunoassays on an Elecsys E-170 analyzer (Roche Diagnostics Systems, Basel, Switzerland). The sensitivity of the method defined as the detectable concentration was 0.15 pg/ml.

### Lentivirus injection

**Figure 2:**
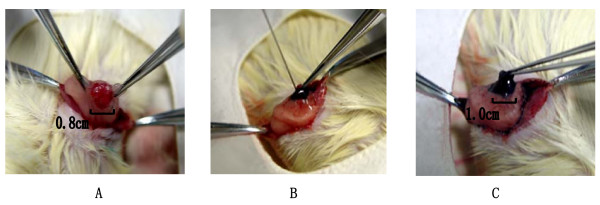
**The surgical diagrams of rat ovarian subcapsular injection and the injection site was seen blue (as injection of mthylene blue)**. A, The rat ovary was presented before injection. B, Mthylene blue was subcapsularly injected into rat ovary. C, The rat ovary was presented after injection of mthylene blue.

Lentivirus was injected subcapularly into the rat ovary with a 10 μl-syringe (Gaoge, Shanghai, China). Needle was injected slowly and held in place for 5 minutes. Each ovary was injected twice on different sites with 10 μl each time. In total, each rat was injected with 40 μl of lentivirus (Fig. 2).

### Fluorescence imaging

Two weeks after lentivirus injection, rat ovaries were removed and cut into 5 μm in thickness of serial sections. GFP fluorescence was observed under microscope using a fluorescence light source and a 485-nm filter (Leica, Germany).

### Statistics

Data were expressed as mean ± SD and analyzed using ANOVA by SPSS 11.0 software (SPSS Inc. Illinois). *p *≤ 0.05 was considered statistically significant.

## Results

### Screen of lentivirus CYP17 shRNA in vitro

**Figure 3:**
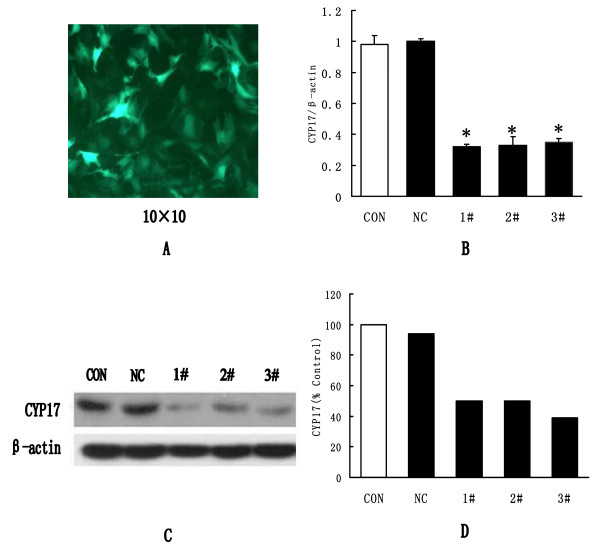
**The CYP17 silenced by lentivirus shRNAs in vitro**. A, GFP fluorescence observed 72 hours after lentivirus transduction. B, Quantitative assay of CYP17 mRNA levels 5 days after lentivirus transduction from six different replicates. C, CYP17 protein levels 7 days after lentivirus transduction. D, Quantitative results of Western blotting images from three different replicates. CON: Blank Control; NC: Negative Control: 1#: CYP17 shRNA1#; 2#: CYP17 shRNA2#; 3#: CYP17 shRNA3#. ANOVA, *P < 0.05 when compared with CON and NC groups. *ANOVA, *P *< 0.05, when compared with both CON and NC groups. CON: Blank Control; NC: Negative Control: 2#: CYP17 shRNA2#.

Three constructed lentivirus shRNAs, targeting different sites of CYP17, were transduced into TIC, cultured in vitro, with a multiplicity of infection of 10 transducing units/ml. Negative and blank control groups were established in the meantime. Seventy-two hours after transduction GFP fluorescence could clearly be seen under fluorescence microscope (Fig 3A). Five days after transduction, cells were harvested and total RNA was extracted for CYP17 mRNA measurement. It showed that all three constructed lentivirus shRNAs had silencing effects on CYP17, and their silence efficiency was 65.5%, 73.7% and 77.4%, respectively (Fig 3B). Seven days after transduction, total protein was extracted for CYP17 protein measurement. Results showed that three constructed lentivirus shRNAs also had silencing effects on CYP17 protein expression, and their silence efficiencies were 50%, 50% and 61%, respectively (Fig. 3C).

For further confirmation of the biological effect of constructed lentivirus CYP17 shRNAs, culture medium was collected for the measurement of androstenedione levels on days 6, 8, and 10 of lentivirus infection (Table 1). On day 6, androstenedione in RNAi group declined slightly, but it declined significantly on day 8. Although androstenedione in the 2# group on day 10 stepped up slightly, compared with day 8, it actually decreased more profoundly compared with the control groups.

**Table 1:** Comparison of androstenedione levels on different days after CYP17 shRNA 2# transduction, values are expressed as mean ± SD.

Lentivirus transduction day	CON (ng/ml) n = 6	NC (ng/ml) n = 6	2# (ng/ml) n = 6
Day 6	0.33 ± 0.028	0.16 ± 0.022	0.15 ± 0.045
Day 8	0.15 ± 0.029	0.081 ± 0.0068	0.047 ± 0.016 *
Day 10	0.32 ± 0.068	0.19 ± 0.078	0.085 ± 0.0094 *

*ANOVA, *P *< 0.05, when compared with both CON and NC groups. CON: Blank Control; NC: Negative Control: 2#: CYP17 shRNA2#.

Through in vitro experiments, we relatively confirmed the silencing effects of constructed lentivirus shRNAs on CYP17 in mRNA, protein and hormone levels. The results showed that all three constructed lentivirus shRNAs had silencing effects on CYP17. We finally chose lentivirus CYP17 shRNA2# for the in vivo animal study because of its consistent effect.

### Delivery efficiency measurement

**Figure 4:**
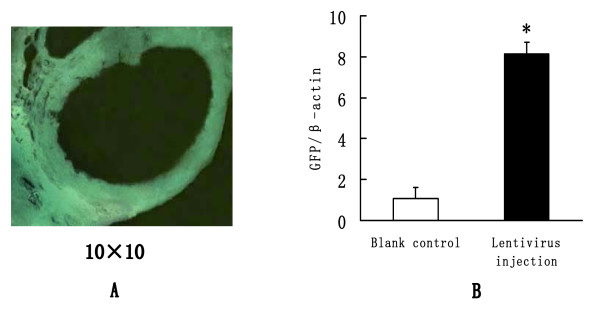
**The delivery efficiency of lentivirus after ovarian capsular injection**. A, The GFP fluorescence of frozen ovarian sections 2 weeks after lentivirus injection. B, Quantitative assay of relative expression level of GFP DNA 2 weeks after lentivirus injection from six different replicates. *Student's t test, *P *< 0.05, when compared with blank control group.

Two weeks after rat ovarian subcapsular injection of lentivirus, animals were sacrificed. Ovarian tissues were removed and frozen sections were prepared. The GFP fluorescence could clearly be seen under fluorescence microscope (Fig. 4A). Ovarian total RNA was also extracted for GFP mRNA level measurement. It showed that the GFP DNA level increased significantly, and its relative expression level was 7.42 times higher than that in the blank control group (Fig. 4B). These results demonstrated that the lentivirus had been successfully delivered to the ovarian tissue by subcapsular injection.

### CYP17 silence in vivo

**Figure 5:**
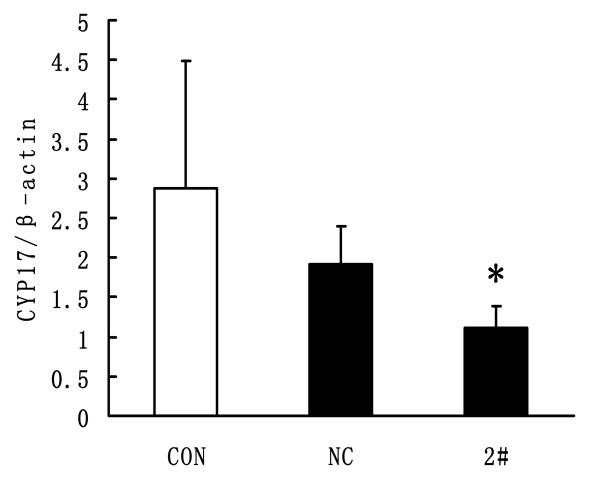
**The relative expression levels of CYP17 mRNA 2 weeks after lentivirus injection from six different replicates**. CON: Blank Control: NC: Negative Control: 2#: CYP17 shRNA2#. ANOVA, *P < 0.05, when compared with both CON and NC groups.

Two weeks after lentivirus CYP17 shRNA2# injection, total RNA of ovarian tissue was extracted for the measurement of silence efficiency of lentivirus shRNA. When compared with the blank control group, CYP17 mRNA levels in the negative control group declined slightly. However, CYP17 mRNA levels declined significantly in the RNAi group with an average inhibition rate of 61% (Fig. 5). Hormone assay results showed that all the levels of androstenedione, 17-hydroxyprogesterone and testosterone declined to a certain degree, but with no statistical significant differences. However, progesterone levels declined significantly (Table 2).

**Table 2:** Comparison of androstenedione 17-hydroxyprogesterone, testosterone and progesterone levels 2 weeks after lentivirus injection.

Ovarian hormones	CON (ng/ml) n = 6	NC (ng/ml) n = 6	2# (ng/ml) n = 6
Androstenedione	0.43 ± 0.19	0.94 ± 0.012	0.33 ± 0.076
17-hydroxyprogesterone	2.56 ± 0.44	1.94 ± 0.18	1.76 ± 0.13
Testosterone	3.14 ± 0.23	3.63 ± 0.23	2.85 ± 0.13
Progesterone	31.52 ± 2.96	34.98 ± 2.68	20.41 ± 1.02*

Values are expressed as mean ± SD

*ANOVA, *P *< 0.05 when compared with both CON and NC groups. CON: Blank Control; NC: Negative Control: 2#: CYP17 shRNA2#.

## Discussion

Androgen excess is the primary pathogenesis of PCOS, in addition, the accentuation of 17alpha-hydroxylase/17, 20-lyase caused by transcriptional augmentation of CYP17 is the most important mechanism [22]. For the first time, we chose CYP17 as the therapeutic target using RNAi technology, silencing the expression of CYP17, and partly inhibiting the ovarian biosynthesis of androgen.

In our study, we constructed three lentivirus shRNAs targeting CYP17. For confirmation of their effectiveness, we transduced lentivirus shRNAs into TIC cultured in vitro, and proved that all three constructed lentivirus shRNAs could silence the expression of CYP17 to different degrees. Through collection of culture medium at different time points for androstenedione determination, it was found that androstenedione began decreasing on day 6, and declined more profoundly on day 8 and day 10. The results suggested that it may be a slow process for lentivirus to integrate into the cellular genome to develop its biological effect. The androstenedione level on day 10 was slightly higher than those on day 6 and day 8, but androstenedione levels in the negative control group on day 10 were also higher than those on days 6 and 8. The explanation for this was that the cell number of TIC on day 10 was increased, and so was the hormone secretion. However, androstenedione still declined more significantly when compared with negative and control groups.

During the in vivo study, we first studied the biological effect of lentivirus CYP17 shRNA on the rat ovary using ovarian subcapsular injection. This method was not only less invasive to the ovary, but it also made lentivirus disperse and sufficiently contact with the follicles, which was beneficial for lentivirus to take effect. The fluorescence and qPCR of GFP also confirmed that the injected lentivirus was successfully delivered into the rat ovary. After injection, CYP17 mRNA declined to a certain degree in the negative control group, which may have been caused by non-specific effects of shRNA. But in the RNAi group the CYP17 mRNA was decreased more significantly, which suggested the shRNA still had a specific silencing effect on CYP17. Two weeks after lentivirus injection, all the levels of androstenedione, 17-hydroxyprogesterone and testosterone declined, but with no significant statistical differences. The possible reasons may be as follows: The dose of injected lentivirus shRNA in vivo was low. It may need to be increased for better silencing effect in vivo. In addition, the small sample number was also a possible reason. Noticeably, progesterone was also decreased, which was similar to our previous study [23]. The possible explanation for this is that the lenivirus shRNA may interfere with the cholesterol uptake and metabolism of TIC, but this notion needs further study to confirm.

## Conclusion

According to our study, ovarian androgen biosynthesis can be partly inhibited by silencing the expression of CYP17. This may not only provide a new trend for the therapy of hyperandrogenism diseases like PCOS, but also set an example for the use of RNAi technology in endocrine diseases. However, there are some limits in our study, such as the safety of lentivirus vectors and the complication of surgical delivery. Moreover, the dose-effect relationship of CYP17 silencing in vivo and its synthetic effect on the metabolism of steroid hormones needs to be further studied.

## Abbreviations

PCOS: Polycystic Ovary Syndrome; RNAi: RNA interference; TIC: theca interstitial cells.

## Competing interests

The authors declare that they have no competing interests.

## Authors' contributions

LY performed most of the experiments, contributed to analysis and interpretation of the data and the writing of the manuscript. LX performed part of the study, coordinated the experiments, contributed to analysis and interpretation of the data. WL, XY and YX assisted in the study. SH and YZ performed the hormone assay. All above-mentioned authors read and approved the final manuscript.
